# Do mitochondria use efflux pumps to protect their ribosomes from antibiotics?

**DOI:** 10.1099/mic.0.001272

**Published:** 2023-01-18

**Authors:** Md Deen Islam, Brian D. Harrison, Judy J.-Y. Li, Austein G. McLoughlin, Deborah A. Court

**Affiliations:** ^1^​ Department of Microbiology, University of Manitoba, Winnipeg, MB, R3T 2N2, Canada

**Keywords:** ABC transporters, MFS transporters, mitochondrial ribosomes, mitochondria drug efflux, *Neurospora crassa*, *Saccharomyces cerevisiae*

## Abstract

Fungal environments are rich in natural and engineered antimicrobials, and this, combined with the fact that fungal genomes are rich in coding sequences for transporters, suggests that fungi are an intriguing group in which to search for evidence of antimicrobial efflux pumps in mitochondria. Herein, the range of protective mechanisms used by fungi against antimicrobials is introduced, and it is hypothesized, based on the susceptibility of mitochondrial and bacterial ribosomes to the same antibiotics, that mitochondria might also contain pumps that efflux antibiotics from these organelles. Preliminary evidence of ethidium bromide efflux is presented and several candidate efflux pumps are identified in fungal mitochondrial proteomes.

## Introduction

Fungi reside in environments such as soils that are rich in antimicrobial compounds produced by bacterial, fungal and archaeal members of the surrounding community [1]. In addition, they must respond to natural and engineered antimicrobials that have entered native, agricultural and host environments due to human activity [2]. Due to selective pressure from the frequent and prolonged use of antimicrobials, it is likely that antimicrobial resistance (AMR) in fungi is increasing, which may lead to potential serious burdens on health care and agricultural production [3, 4]. In bacteria, efflux pumps are responsible for much of the AMR seen, and fungal genomes are rich in coding sequences for these transporters (Table 1), suggesting that they are an intriguing group in which to search for evidence of antimicrobial efflux pumps in mitochondria. In this insight review, we briefly describe the protective mechanisms used by fungi against antimicrobials, and hypothesize that, based on the similarities of their ribosomes to those of Gram-negative bacteria, mitochondria could contain efflux pumps that remove ribosome-targeting antibiotics from these organelles.

**Table 1.** Transporters annotated in genome sequences from representatives of major fungal phyla Numbers were obtained from MycoCosm [87].

Organism and strain	Phylum (taxon)	MFS* (2 .A.1)	DHA1* MDR (2 .A.1.2)	DHA2* MDR (2 .A.1.2)	ABC* (3 .A.1)	ABC – drug resistance†	Genome reference
*Allomyces macrogynus* ATCC 38327	Blastocladiomyetes (Blastocladiomycota)	75	16	7	162	108	Broad Institute
*Chytridium lagenaria* Arg66 v1.0	Chytridiomycetes (Chytridiomycota)	35	5	5	104	74	[88]
*Coemansia mojavensis* RSA 71 v1.0	Kickxellomycotinia (Zoopagomycota)	68	4	14	69	54	[89]
*Rhizophagus clarus* HR1	Glomeromycotina (Mucoromycota)	49	12	3	47	20	[90]
*Aspergillus nidulans*	Eurotiomycetes (Ascomycota)	355	70	33	57	39	[91]
*Neurospora crassa* OR74A V2.0	Sorariomycetes (Ascomycota)	134	23	15	35	39	[92]
*Saccharomyces cerevisiae* S288c	Saccharomycetes (Ascomycota)	136	26	13	34	19	[93]
*Candida albicans* SC5314	Saccharomycetes (Ascomycota)	88	18	6	27	16	[94]
*Schizosaccharomyces pombe*	Schizosaccharomycetes (Ascomycota)	66	24	8	25	10	[95]
*Rhodosporidium toruloides* NP11	Microbotryomycetes (Basidiomycota)	134	34	9	31	22	[96]
*Ustilago maydis* 521 v2.0	Ustilaginomycetes (Basidiomycota)	87	19	6	36	23	[97]
*Agaricus bisporus var bisporus* (H97) v2.0	Agaricomycetes (Basidiomycota)	124	40	30	37	20	[98]

*MFS, Major Facilitator Superfamily; DHA1, Drug:H^+^ antiporter-1; DHA2, Drug:H^+^ antiporter-2; ABC, ATP Binding Cassette family; MDR, multi-drug resistance.

†Total from TCDB 3 .A.1.208, 3 .A.1.201, 3 .A.1.204, 3 .A.1.205 and 3 .A.1.208.

### Antifungal targets and resistance mechanisms

The majority of antifungal compounds used in medicine target molecules and metabolic pathways that are specific to these organisms, while others that target mitochondria are more commonly used in agriculture (reviewed by [5, 6]). Below we describe some examples of antifungal compounds, their targets and resistance mechanisms.

### Cell wall- and sterol-specific targets and resistance

Compounds frequently used to combat fungal infections in animals include polyenes, such as amphotericin B, that disrupt the integrity of the fungal cell membrane by interacting with ergosterol. Triazoles (fluconazole, prothioconazole) inhibit the 14-α-demethylase, which converts lanosterol to 4,4′-dimethyl cholesta-8,14,24-triene-3-beta-ol, blocking ergosterol synthesis and concomitantly causing the accumulation of toxic 14-methylsterols. Echinocandins block 1,3-β-d-glucan synthase, increasing the permeability of the fungal cell wall [7]. Chitin synthase and the GPI-anchored wall protein transfer polypeptide (Gwt1) from the glycosylphosphatidylinositol (GPI) anchor pathway are other cell wall synthesis targets under investigation (reviewed by [8]). Nucleotide analogues including 5-fluorocytosine (5-FC) interfere with pools of deoxyribonucleotides and can interfere with protein synthesis and reduce levels of thymine deoxyribonucleotides [6, 9].

Resistance to antifungals can result from target modification or overexpression, changes in target activity, drug detoxification, efflux of the drug either from the cell or into vacuoles, regulation of metabolism, and/or stress response pathways (reviewed by [10]). For example, increased expression of the sterol 14-α demethylase (*ERG11*) gene in *Candida albicans* [11] and single amino-acid substitutions in that protein [12] are associated with azole resistance. In another example, deletion of the gene for one of the vacuolar efflux pumps Vba1, Vba2 or Vba4 increases the susceptibility of *Saccharomyces cerevisiae* to azoles, implicating the sequestration of antifungals in vacuoles as part of cellular resistance [13]. Furthermore, it is important to note that efflux pump-mediated resistance is the most prominent antifungal resistance mechanism and often leads to multi-drug resistance (MDR [14, 15]). In fungi, increased expression of efflux pumps, of either the ATP Binding Cassette (ABC) or Major Facilitator Superfamily (MFS) type, in the plasma membrane is associated with MDR (reviewed by [16, 17]). These pumps will be described in more detail in a subsequent section.

### Electron transport chain targets and resistance

Antifungal agents applied to plants include several with mitochondrial targets. The succinate dehydrogenase inhibitors (SDHIs) inhibit complex II of the mitochondrial respiratory chain [18] and anilinopyrimidines target mitochondrial pathways, as resistance can be associated with alleles of multiple genes whose products function in mitochondria, including the NADH kinase [19]. The strobilurins inhibit respiration through interactions with the quinol oxidation site of cytochrome *b*, thereby blocking electron flow through complex III and reducing the development of the proton motive force required for ATP generation [20]. Resistance to these agents can occur through point mutations in the target proteins (reviewed by [21]) and MDR can result from overexpression of ABC [22] or MFS [23] efflux pumps. There is no evidence for resistance through overexpression of electron transport chain (ETC) subunits, which is not unexpected as they function in large multi-subunit complexes of defined stoichiometry.

### DNA and protein biosynthesis targets and resistance

The nucleoside analogue 5-FC has a long history of use as an antifungal agent [24], both alone and in combination with amphotericin B [25]. The 5-FC prodrug is metabolized by fungal enzymes from the pyrimidine salvage pathway to produce intermediates that can interfere with thymidylate synthase activity, and can be incorporated into RNA, rendering it non-functional. Resistance to 5-FC occurs frequently and is often associated with mutations that decrease expression or lead to non-functional enzymes in the pyrimidine salvage pathway (reviewed in [26]).

### Mitochondrial ribosomal targets and resistance

Numerous antibacterial compounds prevent translation through interactions with ribosomes. Mitochondria share a common ancestor with bacteria [27], and their 70S ribosomes are also susceptible to many of the same drugs. Antibacterials such as chloramphenicol, tetracycline and erythromycin block mitochondrial translation (Table 2) and thereby prevent or inhibit growth of obligately aerobic fungi on respiratory substrates by blocking synthesis of mitochondrially encoded subunits of ETC or ATP synthase. In *Saccharomyces cerevisiae*, these essential proteins include apocytochrome *b* [28], subunits of cytochrome oxidase [29] and ATP synthase [30], as well as, in some yeasts, a subunit of the mitochondrial ribosome [31], or subunits of NADH dehydrogenase [30]. Partial inhibition of respiration in plants and animal cells is associated with signalling from the stress pathways responding to unfolded mitochondrial proteins and increased levels of reactive oxygen species, both of which are associated with mitochondrial dysfunction [32]. Translation-inhibiting antimicrobials are not routinely used in medicine or agriculture to treat fungal infections, due to the high concentration of drug needed for antifungal activity (Table 2) and the sensitivity of host mitochondria to the drugs.

**Table 2.** Sensitivity of yeast to mitochondrial targeting antibiotics

Antibiotic (class)	Target and mechanism	Inhibition of respiratory growth	Inhibition of mt-translation (% inhibition)	MIC* E. coli (strain, reference)
Chloramphenicol	Binds the large subunit rRNA Prevents entry of aminoacyl-tRNA into the A site [99]	4000 µg ml^−1^ [100]	9.7 µg ml^−1^ (80–90 %) [101]	6.3 µg ml^−1^ (W3110, [102]) 8 µg ml^−1^ (CP78, [103])
Erythromycin (macrolide)	Binds large subunit rRNA at the nascent peptide exit tunnel Prevents peptide bond formation at certain amino acid sequences [99]	300–1000 µg ml^−1^ [104]	0.7 µg ml^−1^ [104]	50 µg ml^−1^ (W3110, [102])
Kanamycin (aminoglycoside)	Binds small subunit rRNA Inhibits activity of the decoding centre in the A site [105]	>4800 µg ml^−1^ [101]	9.7 µg ml^−1^ (80–90 %) [101]	32 µg ml^−1^ (CP78, [103])
Tetracycline	Binds small subunit Inhibits binding of aminoacyl-tRNA at the A site [99]	100–3000 µg ml^−1^ [106]	No data	1.25 µg ml^−1^ (W3110, [102])
Paromomycin (aminoglycoside)	Binds small subunit rRNA Inhibits activity of the decoding centre in the A site [99]	2000 µg ml^−1^ [107]	31 µg ml^−1^ (~80 %) [101]	10 µg ml^−1^ (SK901, [108])

*MIC, minimum inhibitory concentration for *Escherichia coli*.

**Fig. 1.**
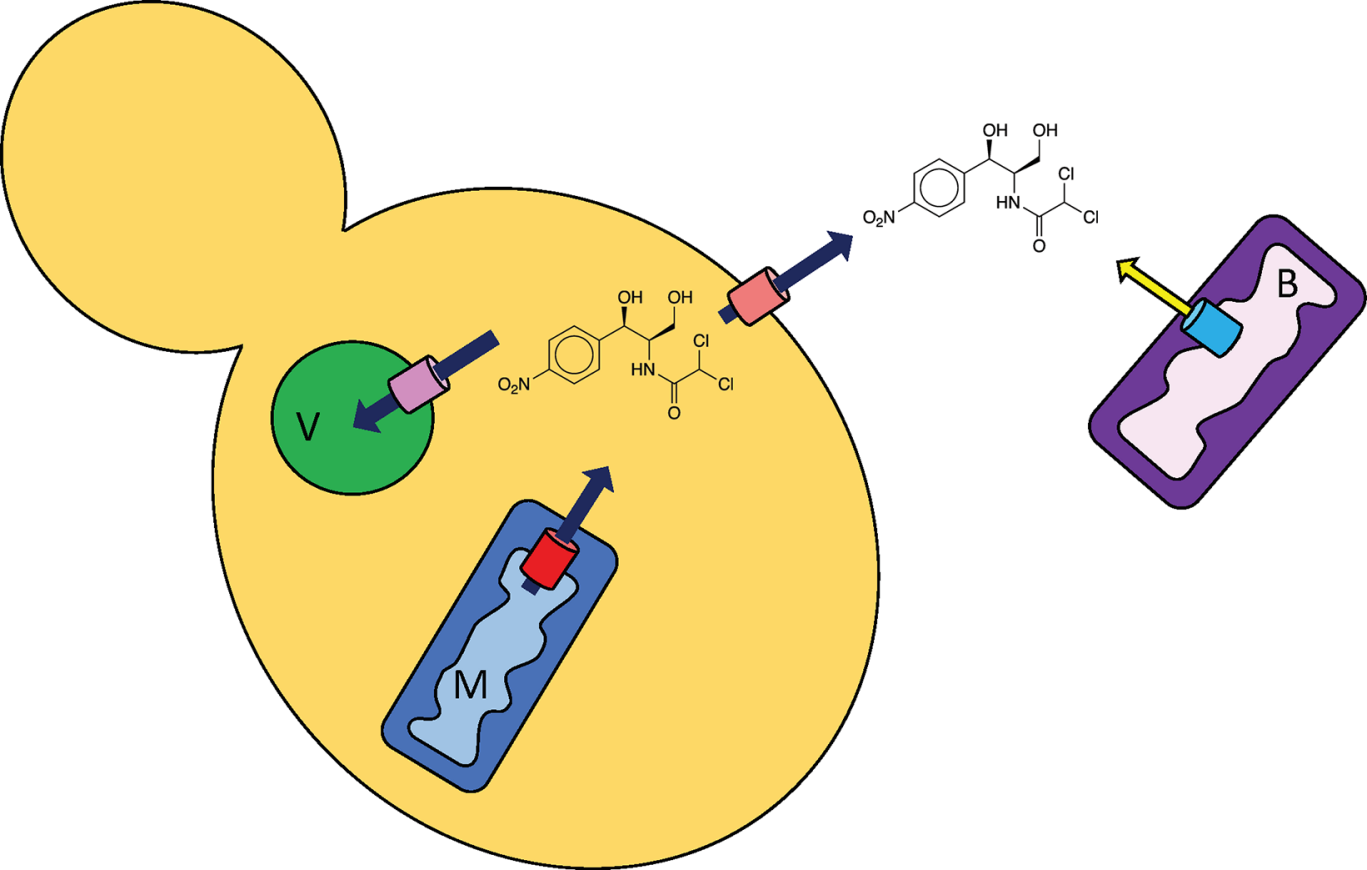
Illustration of potential mechanisms of efflux of antimicrobials in a yeast cell. Efflux pumps are shown as cylinders in the vacuolar (V), mitochondrial (M) and plasma membranes. In comparison, efflux from a Gram-negative bacterium (B) is shown. In bacteria and mitochondria (M), the pumps reside in the inner/periplasmic membrane. Chloramphenicol is shown as an example. See text for details.

In fungi, heritable resistance to erythromycin [33] and chloramphenicol [34, 35] can occur through single point mutations in the mitochondrial rRNA genes, as demonstrated in parallel studies in bacteria (reviewed by [36]). This is unlikely to be the only mechanism for protecting mitochondrial translation because intact fungi tolerate much higher concentrations of drug than are needed to inhibit translation in isolated organelles (Table 2). For example, chloramphenicol at 500 µg ml^−1^ inhibits mitochondrial (mt)-translation *in vivo*, while about 10 µg ml^−1^ is sufficient in isolated organelles; the latter value is in the range of the MIC for *Escherichia coli* (~6 µg ml^−1^; Table 2). This suggests that one or a combination of the following mechanisms maintain a low intracellular drug concentration: limited access to the cell due to cell wall components, compartmentalization in vacuoles, and efflux across the plasma and mitochondrial membranes (Fig. 1). Efflux pumps can be unique to one membrane, or localized to more than one site. For example, Tpo1, a polyamine efflux pump in *Saccharomyces cerevisiae* that participates in cycloheximide, quinidine [33] and caspofungin [37] resistance, is localized to the plasma membrane [38], vacuolar membranes [39] and the mitochondrial inner membrane (MIM [36, 40]).

### Preliminary evidence for drug efflux from fungal mitochondria

To the best of our knowledge, evidence for mitochondrial MDR efflux pumps is limited to those in the human breast carcinoma cell line MCF-7S, which displays doxorubicin (DOX) resistance linked to the ABC mitochondrial drug efflux pump, breast cancer resistance protein BCRP1 (ABCG2) and the MDR-associated protein MRP1 (ABCC1 [41]. DOX, used in cancer treatment due to its inhibition of nuclear topoisomerases, is associated with dysfunctional mitochondria with increased susceptibility to apoptosis [42]. While these observations confirm that drug efflux can impact mitochondria, the drug in this case does not target mitochondrial translation.

**Fig. 2.**
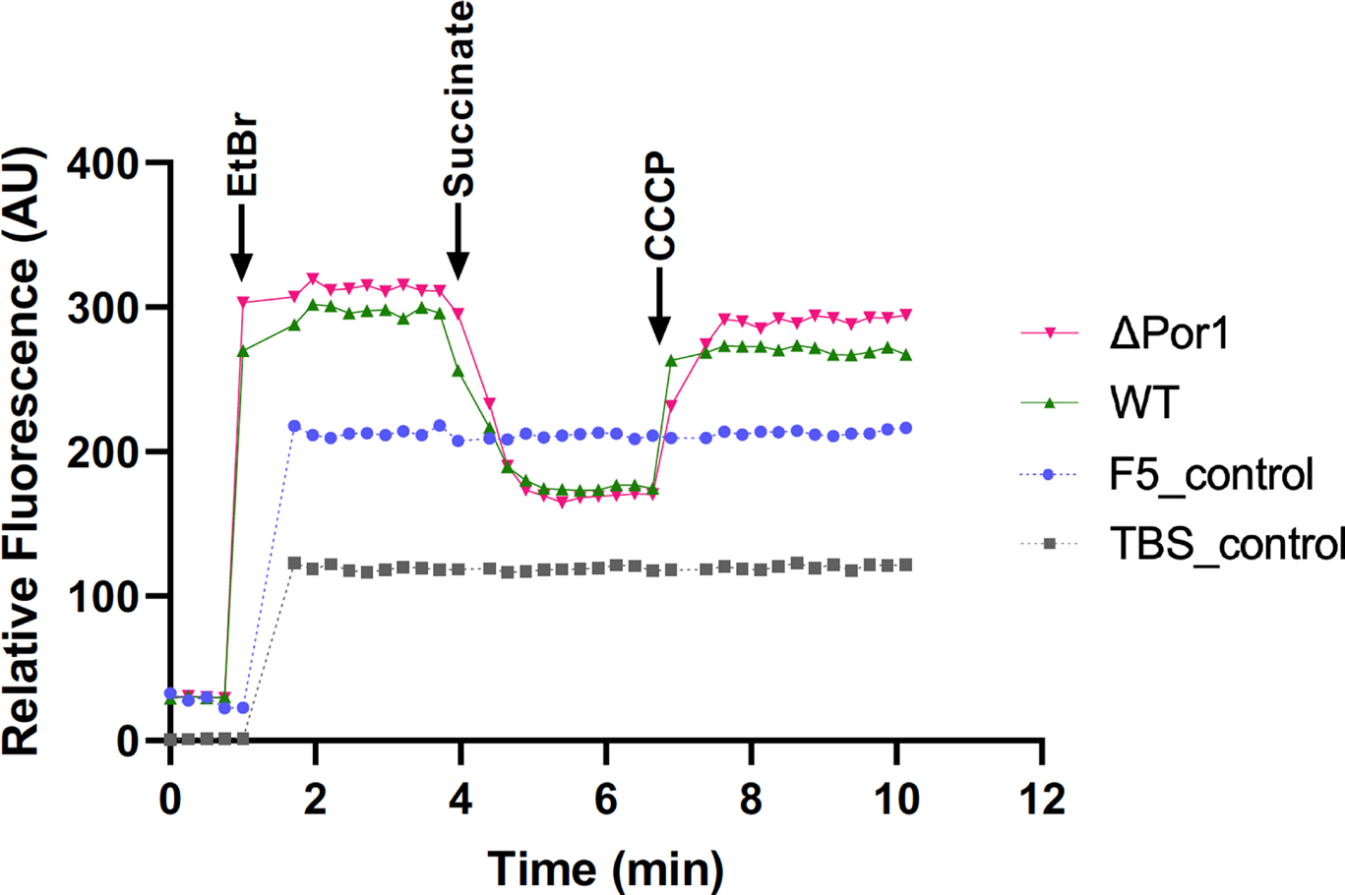
Mitochondrial EtBr efflux assays. Mitochondria were prepared as described [48]. The 2 ml reactions contained 0.24 mg ml^−1^ mitochondrial protein in buffer (250 mM sucrose, 30 mg ml^−1^ BSA, 5 mM MgCl_2_, 80 mM KC1, 10 mM MOPS, pH 7.2). After approximately 2 min, 10 µl of 1 mg ml^−1^ EtBr was added to each cuvette, followed by stepwise additions of 10 µl of 1 M succinate, 2 µl of 1 mM oligomycin and 10 µl of 2 mM CCCP. The assay was carried out at 25 °C, with continuous stirring, in an Agilent Cary Eclipse fluorescence spectrophotometer (excitation 520 nm and emission 590 nm; slit width 5 nm). The fluorescence was allowed to stabilize between each addition and fluorescence was measured every 15 s. WT, *N. crassa* wild-type (FGSC 9718); ΔPor1, an isogenic porin deletion strain [50]. F5 is the buffer control and TBS (Tris-buffered saline) is a buffer control lacking BSA. Taken from [109], with permission.

As a first step in determining the potential for mitochondrial drug efflux in fungi, ethidium bromide (EtBr) efflux assays were used. EtBr is a common substrate for assays of ABC [43], MFS [44], MATE (multidrug and toxin extrusion) [45], SMR (small multidrug resistance) and RND (resistance-nodulation-cell division) [46] pump activity in bacteria. To assess whether isolated mitochondria are capable of efflux, they were prepared from *Neurospora crassa,* and used in an efflux assay (Fig. 2 [43, 47]). Mitochondria, depleted for membrane potential, were incubated with EtBr, which is taken up and intercalated into DNA, thereby increasing its fluorescence. A membrane potential was generated by the addition of succinate [48], which could activate efflux pumps of the MFS. This led to a sharp decrease in fluorescence, suggesting the presence of proton-drug antiporters. ADP was not included in the assay buffer, precluding the synthesis of ATP. Dissipation of the membrane potential by the addition of the uncoupler carbonyl cyanide m-chlorophenyl hydrazone (CCCP) led to an increase in fluorescence, suggesting that EtBr was no longer being expelled from the organelles. Together, these observations are compatible with efflux of EtBr by proton-motive force-driven pump(s) from the MFS, RND, SMR [46] or PACE (proteobacterial antimicrobial compound efflux) [49] families. These effects were seen in mitochondria from a strain lacking the major outer membrane (MOM) porin, VDAC [50], which is suggestive of pump(s) that do not require an outer membrane component or use a different pore in the MOM.

## Transporters annotated in fungal genomes

### Classes of drug efflux proteins

With preliminary evidence for efflux from fungal mitochondria, the goal was to search fungal genomes and mitochondrial proteomes for evidence of potential efflux pumps. Six families of proteins are involved in drug efflux [51, 52]: the ABC family, which utilizes ATP hydrolysis to drive transport (see below); the MFS family (see below); the MATE family, which includes NorM from *Vibrio parahaemolyticus* [53]; the SMR family, transporters of around 100 residues, such as QacC of *Staphylococcus aureus* [54]; the large multi-component, RND superfamily, including AcrB of *E. coli* [55]; and the more recently recognized PACE family, which includes the *Acinetobacter baumannii* AceI protein [56]. For this discussion, we focused on the two large families of pumps involved in drug efflux in bacteria, and in the plasma and vacuolar membranes of eukaryotes, namely the ABC and MFS transporters.

ABC transporters (Transporter Classification Data Base, TCDB, https://www.tcdb.org/, ID 3 .A.1 [57]) include multiple subfamilies involved in import and export of substrates ranging from antimicrobials to heavy metals and toxins [58]. An ABC transporter consists of two transmembrane domains, each formed by hydrophobic transmembrane α-helices (TMH) and two nucleotide binding domains (reviewed in [59, 60]). A single polypeptide may comprise all four domains, or they may be found on two polypeptides, in which case the functional form is a homo- or heterodimer [59]. These proteins shuttle substrates across membranes using the ‘alternative access’ model, in which substrate binds an inwardly facing open state when the transporter is bound to ATP. Subsequent hydrolysis of ATP converts the structure to an outwardly facing open state, allowing release of substrate [60]. Bacterial ABC proteins involved in antibiotic efflux are usually members of tripartite structures comprising the ABC protein, a periplasmic component and an outer membrane pore that allow efflux from the periplasm, or from the cytoplasm, across the inner membrane, and to the exterior of the cell (reviewed in [52]).

The MFS is a very old, large and diverse superfamily of uniporters, antiporters and symporters that utilize the electrochemical gradient of protons to transport substrates across membranes [61]. The family currently includes 89 subfamilies of proteins in bacteria, eukaryotes and archaea (reviewed in [62], TCDB ID 2 .A.1) [57]. Members of this family catalyse uniport, solute:cation (H^+^) symport and/or solute:H^+^ or solute:solute antiport. MFS permeases exhibit specificity for sugars, polyols, drugs, Krebs cycle metabolites, phosphorylated glycolytic intermediates, amino acids, peptides, osmolytes, siderophores (efflux), iron-siderophores (uptake), nucleosides, and organic and inorganic anions. The MFS proteins use a ‘rocker-switch’ mechanism that transports substrates across the membrane via a series of conformational changes resulting from coupled binding of protons and substrates to the transporter [63]. MFS proteins are about 400–600 aa long and consist of TMH. Most MFS associated with drug resistance can be classified into the drug:H^+^ antiporter-1 (DHA1) family (12 TMH; TCDB ID 2 .A.1.2) or the DHA2 family (14 TMH, TCDB ID 2 .A.1.3) [62, 64]. DHA2 proteins are found in tripartite complexes, whereas DHA1 transporters function as single proteins [52].

### Fungal genomes

To determine the prevalence of these classes of potential drug transporters in fungi, annotations of genomes from organisms representing the major fungal phyla [65] were scanned for transporters of the two major classes: MFS (TCDB 2 .A.1) and ABC (3 .A.1) (see Table 1 and references therein). In these fungi, the number of MFS-coding genes ranges from 35 in *Chytridium lagenaria*, representing an early diverging fungal lineage, to over 350 in *Aspergillus nidulans*. The number of ABC transporter genes ranges from around 30 in the representative ascomycetes and basiomycetes to 162 in *Allomyces macrogynus*, also an early branching fungal group [65]. Between 25 and 55 % of the MFS pumps predicted in this sample of genomes belong to either the DHA1 or DHA2 family; in most cases the number of DHA1 exceeds that of DHA2. Lists of predicted ABC and MFS transporters for two model fungi, *Saccharomyces cerevisiae* and *N. crassa*, are found in Tables S1 and S2 (available with the online version of this article).

There are several subfamilies of ABC transporters involved in drug resistance; they fall into the following families: (Putative) Drug Resistance ATPase-2 (Drug RA2, TCDB 3 .A.1.208), Multidrug Resistance Exporter (MDR; ABCB; 3 .A.1.201), Eye Pigment Precursor (EPP, 3 .A.1.204), Pleiotropic Drug Resistance (PDR 3 .A.1.205) and Drug Conjugate Transporters (DCT, 3 .A.1.208) [57]). Between 40 and 78 % of the predicted ABC transporters in each organism in this sample fall into one of these families (Table 1). The range in numbers of transporters per organism may reflect the fact that, in addition to drug efflux, MFS members are involved in many processes including secretion of antimicrobials and secondary metabolites produced by fungi [66]. In addition, other MFS proteins participate in compartmentalization of intermediates in peroxisomes, where enzymes involved in the antibiotic biosynthetic pathways reside. For example, in *Penicillium chrysogenum* peroxisomal MFS transporters are involved in importing penicillin precursors into the peroxisome [67], while ABC transporters are predicted to be involved in secretion [68]. In some cases, such as AtrD of *P. chrysogenum*, the same pump may be involved in secretion of antibiotics generated by the organism and efflux of others encountered from the environment [66].

### Transporters in mitochondrial proteomes

The vast majority of the 1000–1500 proteins in mitochondria are encoded by nuclear genes, translated in the cytoplasm and imported into the organelle (reviewed by [69]). Proteins destined for the mitochondrial matrix, as well as a subset of those in the MIM and intermembrane space, contain canonical N-terminal signal sequences, allowing prediction of their mitochondrial localization (ex. [70]). However, with a few exceptions such as the ADP/ATP carrier (AAC [71]), the targeting information for inner membrane proteins has yet to be well delineated, precluding *in silico* prediction of mitochondrial MFS or ABC transporters. Therefore, to identify putative efflux pumps, we searched published mitochondrial proteomes for proteins related to MFS or ABC MDR transporters in *N. crassa* and *Saccharomyces cerevisiae*, and for genes identified in screens of yeast deletion libraries for increased sensitivity to antibiotics.

### Proteomes

Mitochondrial proteomes from *Saccharomyces cerevisiae* [40, 72, 73], covering 1221 unique proteins (Fig. S1), were compared to the list of annotated MFS and ABC transporters in Table S1. Nine transporters were detected in at least one of the proteomes (Table 3). Of the ABC transporters, three are localized to the MIM: the well-characterized iron–sulphur cluster transport protein Atm1 [74], and the two MDR-like transporters Mdl1 and Mdl2. Mdl1 was identified as a peptide transporter [75] and is associated with resistance to a variety of compounds including doxorubicin (Table S3). The related protein Mdl2 is associated with increased resistance to two mitochondrial targeting drugs: strobilurin B and antimycin A (Table S3). The oligomycin-resistance ATP-dependent permease, Yor1, has been localized to the plasma membrane and the MIM [40], preventing oligomycin from reaching its mitochondrial target, ATP synthase. The absence of each of these ABC transporters is associated with increased sensitivity to a number of chemicals (Table S3), a few of which have mitochondrial targets. However, no transporters with specificity for drugs that target mitochondrial translation were identified.

**Table 3.** Transporters detected in mitochondrial proteomes of *Saccharomyces cerevisiae*

Gene*	Protein name†	Type of transporter‡	*Candida albicans* (accession no., e-value)§	*Arabidopsis thaliana* (accession no., e-value)§	*Homo sapiens* (accession no., e-value)§	Proteome reference(s)
*ATM1*	Iron–sulphur clusters transporter	ABC||	P97998, 4.3E^−57¶	Q9LVM1, 7.1E^−188	B4DGL8, 8.0 E^−189	[72, 73]
*MDL1*	ATP-dependent permease, multidrug resistance-like protein	ABC	P97998, 9.5E^−183¶	Q9C7F8, 4.0E^−165	P08183, 4.9E^−156	[72, 73]
*MDL2*	ATP-dependent permease, multidrug resistance-like protein 2	ABC	P97998, 7.9E^−153¶	Q9LJX0, 2.6E^−166	P08183, 2.6E^−161	[72, 73]
*YOR1*	Oligomycin resistance ATP-dependent permease	ABC	G1UAZ5, 4.1E^−183#	Q42093, 7.4E^−205	O15439, 3.6E^−213	[73]
*HXT2*	High-affinity glucose transporter	MFS||	KGR01337.1, 0**	P23586, 1 .9E^−54	Q96QE2, 6.5E^−41	[40, 73]
*JEN1*	Carboxylic acid transporter protein homologue	MFS	KGU09375.1, 9E^−121**	Q9SYQ1, 0.00014¶	A0A286YF51, 0.0019	[73]
*TPO1*	Polyamine transporter 1	MFS	G1UAY0, 1.9E^−141#	Q56ZZ7, 0.00017	Q6ZMD2, 9.2E^−6	[40]
*TPO3*	Polyamine transporter 3	MFS	Q9C0R8, 6.1E^−98#	A0A1I9LP99, 7.5E^−5	Q7L0J3, 0.0047¶	[40, 73]

*Information adapted from the *Saccharomyces* Genome Database (SGD). https://www.yeastgenome.org/.

†Information adapted from UniProtKB. https://www.uniprot.org/uniprot/.

‡Information adapted from the Transporter Classification Database (TCDB). https://www.tcdb.org/

§Information gathered from NCBI – Protein BLAST, algorithm: PSI-BLAST (https://www.ncbi.nlm.nih.gov/) and HMMER – phmmer, Database: Reference Proteomes (http://hmmer.org/ *)*. The accession code from UniprotKB and e-value from HMMER-phmmer results were used.

||ABC = ATP Binding Cassette; MFS = Major Facilitator Superfamily.

¶Identified in *Candida albicans* [110], human [110] or *Arabidopsis thaliana* [111] proteomes.

#Information gathered from NCBI – Protein BLAST, algorithm: PSI-BLAST and ^#^HMMER-phmmer, Database: UniProtKB or *HMMER – phmmer, Database: SwissProt. The accession code from UniprotKB and e-value from HMMER-phmmer results were used.

**Exceptions: Information only gathered from NCBI – Protein BLAST, algorithm: PSI-BLAST. The accession code and e-value from PSI-BLAST results were used.

The MFS proteins detected in *Saccharomyces cerevisiae* mitochondrial proteomes included the high-affinity glucose transporter, Hxt2, a homologue of carboxylic acid transporters, and two polyamine transporters, Tpo1 and Tpo3 (Table 3), both of which are found in plasma, vacuolar and mitochondrial membranes and are associated with resistance to multiple chemicals in addition to polyamines (Table S3). Only Tpo3 is known to contribute to resistance to a drug with a mitochondrial target, the uncoupler carbonyl cyanide *p*-trifluoromethoxyphenylhydrazone (FCCP). Protein homology searches using HMMER3 [76] and PSI-Blast [77] revealed that many of these proteins have potential homologues in the clinically important *Candida albicans*, the model plant *Arabidopsis thaliana* and *Homo sapiens* (Table 3), although this is not unexpected given the conservation in the MFS.

Similarly, two proteomes from *N. crassa* [78, 79] were scanned for proteins of the MDR and ABC families and four of each were found (Table 4). Of the MFS proteins, two are annotated as sugar transporters and one as a phospholipid transporter. The fourth (NCU09551) is predicted to be a DHA2-MDR protein; it does not show strong sequence similarity with characterized MDR-MFS proteins in fungi. Four ABC transporters were identified: Atm1, Mdr-3 and two other unnamed ABC transporters (Table 4). Mdr-3 is about twice the size of *Saccharomyces cerevisiae* Tpo1 and its C-terminal half shares about 20 % sequence identity with *Saccharomyces cerevisiae* Tpo1. Mdr-3 in *N. crassa* is associated with resistance to staurosporine, a protein kinase inhibitor [80]. Transcription of both *MDR-3* and the ABC transporter gene NCU07546 is induced in the presence of (thio)xanthones, compounds with anti-cancer properties [81], suggesting that they could be involved in a response to toxins. Large-scale screens for drug sensitivity have not been carried out in *N. crassa*.

**Table 4.** Transporters detected in mitochondrial proteomes of *Neurospora crassa*

Gene, systematic name	SwissProt/UniProtKB	Type of transporter	*Candida albicans* (accession no., e-value)*	*Arabidopsis thaliana* (accession no., e-value)*	*Homo sapiens* (accession no., e-value)*	Reference
NCU07546	Q7RVS1	ABC	P97998, 4.1E^−80†	Q9SYI2, 3.2E^−267	P08183, 4.0E^−298	[79]
*MDR3*, NCU09975	V5IP78	ABC	P97998, 8.0E^−63†	Q9FHF1, 3.9E^−225	P08183, 8.4E^−237	[79]
*ATM1,* NCU05029	Q7RX59	ABC	P97998, 5.2E^−58†	Q9LVM1, 1.0E^−197	O75027, 2.3E^−197	[79]
NCU05226	Q7S8I2	ABC	O42765, 7.8E^−6‡	A0A1P8ATY7, 6.7E^−12	Q8WW24, 1.4E^−14	[79, 112]
*HXT13,* NCU01633	Q1K4S3	MFS	Q8J2J7, 2.1E^−85†	P23586, 1.2E^−52	P11168, 2.6E^−48	[79]
NCU04809	Q756M6	MFS	T2AWG3, 0.0014‡	Q8GYF4, 1.4E^−24	B3KT41, 0.0022	[79]
NCU09551	Q7S2B0	MFS	G1UAY0, 2.3E^−6‡	Q56ZZ7, 2.3E^−7	Q8NBP5, 8.4E^−9	[78]
NCU10021	Q7S0I5	MFS	Q8J2J7, 8.1E^−102‡	Q9FMX3, 2.3E^−55	Q96QE2, 2.8E^−41	[78]

*Information gathered from NCBI – Protein BLAST, algorithm: PSI-BLAST (https://www.ncbi.nlm.nih.gov/) and HMMER – phmmer, Database: Reference Proteomes (http://hmmer.org/ *)*. The accession code from UniprotKB and e-value from HMMER-phmmer results were used.

†Identified in *Candida albicans* [110], human [110], or *Arabidopsis thaliana* [111] proteomes.

‡Exceptions: information gathered from NCBI – Protein BLAST, algorithm: PSI-BLAST and ^#^HMMER-phmmer, TCDB, UniProtKB or ^*^HMMER – phmmer, Database: SwissProt. The accession code from UniProtKB and e-value from HMMER-phmmer results were used.

ABC, ATP - Binding Cassette; MFS, Major Facilitator Superfamily.

### Genes associated with antibiotic sensitivity in *Saccharomyces cerevisiae*

A second approach to identifying potential mitochondrial efflux pumps was to compile a list of *Saccharomyces cerevisiae* genes associated with reduced sensitivity to the drugs tetracycline, erythromycin and chloramphenicol in large-scale deletion screens [82]. Since these three drugs target the mitochondrial ribosome, it was hypothesized that mitochondrial transporters will have evolved to efflux them and therefore protect the mitochondria. A total of 105 genes in *Saccharomyces cerevisiae* have been documented to confer some resistance to at least one of these three drugs and a total of 25 were found in at least one of the mitochondrial proteomes (Table S4). However, only two of the 25 proteins are predicted transporters; they are Yor1 (part of the ABC superfamily, discussed above) and Rim2, a bifunctional iron/pyrimidine nucleotide transporter of the mitochondrial carrier family in mitochondria (TCDB 2 .A.29.10.4 [83]). A *Rim2Δ* strain shows increased sensitivity to erythromycin and tetracycline, while a *Yor1Δ* strain shows increased sensitivity to tetracycline [82], suggesting that these could be involved in drug efflux.

## Concluding remarks

Based on the exposure of fungi to antimicrobials with the potential to impact mitochondrial ribosome function, and the abundance of transporter-encoding genes in these organisms, it is compelling to search for mitochondrial efflux pumps that are part of a network of plasma membrane and vacuolar transporters that together protect the organelle’s translation machinery. An EtBr efflux assay supports the presence of such pumps. Identification of pumps will benefit from increased coverage of membrane proteins in proteomic screens [84], combined with systematic screens of putative pump deletion variants in a variety of model organisms and clinical fungal isolates. Furthermore, efflux from mitochondria may play a role in protecting mammalian mitochondrial function from antibiotics, as well as from antivirals and anticancer agents such as nucleoside reverse transcriptase inhibitors and ribonucleoside-based inhibitors (reviewed by [85]) that can be incorporated by mitochondrial polymerases into the corresponding nucleic acids, causing mitotoxicity and mitochondrial DNA loss [86].

## Supplementary Data

Supplementary material 1

Supplementary material 2

## References

[1] Mullis MM, Rambo IM, Baker BJ, Reese BK (2019). Diversity, ecology, and prevalence of antimicrobials in nature. Front Microbiol.

[2] Cycoń M, Mrozik A, Piotrowska-Seget Z (2019). Antibiotics in the soil environment-degradation and their impact on microbial activity and diversity. Front Microbiol.

[3] Hendrickson JA, Hu C, Aitken SL, Beyda N (2019). Antifungal resistance: a concerning trend for the present and future. Curr Infect Dis Rep.

[4] Brauer VS, Rezende CP, Pessoni AM, De Paula RG, Rangappa KS (2019). Antifungal agents in agriculture: friends and foes of public health. Biomolecules.

[5] Fisher MC, Hawkins NJ, Sanglard D, Gurr SJ (2018). Worldwide emergence of resistance to antifungal drugs challenges human health and food security. Science.

[6] Houšť J, Spížek J, Havlíček V (2020). Antifungal drugs. Metabolites.

[7] Szymański M, Chmielewska S, Czyżewska U, Malinowska M, Tylicki A (2022). Echinocandins - structure, mechanism of action and use in antifungal therapy. J Enzyme Inhib Med Chem.

[8] Lima SL, Colombo AL, de Almeida Junior JN (2019). Fungal cell wall: emerging antifungals and drug resistance. Front Microbiol.

[9] Jørgensen LN, Heick TM (2021). Azole use in agriculture, horticulture, and wood preservation - is it indispensable?. Front Cell Infect Microbiol.

[10] Hu M, Chen S (2021). Non-target site mechanisms of fungicide resistance in crop pathogens: a review. Microorganisms.

[11] Flowers SA, Barker KS, Berkow EL, Toner G, Chadwick SG (2012). Gain-of-function mutations in UPC2 are a frequent cause of ERG11 upregulation in azole-resistant clinical isolates of *Candida albicans*. Eukaryot Cell.

[12] Xiang M-J, Liu J-Y, Ni P-H, Wang S, Shi C (2013). Erg11 mutations associated with azole resistance in clinical isolates of *Candida albicans*. FEMS Yeast Res.

[13] Kawano-Kawada M, Pongcharoen P, Kawahara R, Yasuda M, Yamasaki T (2016). Vba4p, a vacuolar membrane protein, is involved in the drug resistance and vacuolar morphology of *Saccharomyces cerevisiae*. Biosci Biotechnol Biochem.

[14] Cowen LE, Sanglard D, Howard SJ, Rogers PD, Perlin DS (2014). Mechanisms of antifungal drug resistance. Cold Spring Harb Perspect Med.

[15] Holmes AR, Cardno TS, Strouse JJ, Ivnitski-Steele I, Keniya MV (2016). Targeting efflux pumps to overcome antifungal drug resistance. Future Med Chem.

[16] Sá-Correia I, dos Santos SC, Teixeira MC, Cabrito TR, Mira NP (2009). Drug:H+ antiporters in chemical stress response in yeast. Trends Microbiol.

[17] Kim J, Cater RJ, Choy BC, Mancia F (2021). Structural insights into transporter-mediated drug resistance in infectious diseases. J Mol Biol.

[18] Luo B, Ning Y (2022). Comprehensive overview of carboxamide derivatives as succinate dehydrogenase inhibitors. J Agric Food Chem.

[19] Mosbach A, Edel D, Farmer AD, Widdison S, Barchietto T (2017). Anilinopyrimidine resistance in *Botrytis cinerea* is linked to mitochondrial function. Front Microbiol.

[20] Musso L, Fabbrini A, Dallavalle S (2020). Natural compound-derived cytochrome bc1 complex inhibitors as Antifungal Agents. Molecules.

[21] Leroux P, Gredt M, Leroch M, Walker AS (2010). Exploring mechanisms of resistance to respiratory inhibitors in field strains of *Botrytis cinerea*, the causal agent of gray mold. Appl Environ Microbiol.

[22] Sang H, Chang HX, Choi S, Son D, Lee G (2022). Genome-wide transcriptional response of the causal soybean sudden death syndrome pathogen *Fusarium virguliforme* to a succinate dehydrogenase inhibitor fluopyram. Pest Manag Sci.

[23] Omrane S, Sghyer H, Audéon C, Lanen C, Duplaix C (2015). Fungicide efflux and the MgMFS1 transporter contribute to the multidrug resistance phenotype in *Zymoseptoria tritici* field isolates. Environ Microbiol.

[24] Tassel D, Madoff MA (1968). Treatment of *Candida sepsis* and *Cryptococcus meningitis* with 5-fluorocytosine. A new antifungal agent. JAMA.

[25] Mourad A, Perfect JR (2018). Present and future therapy of *Cryptococcus* infections. JoF.

[26] Delma FZ, Al-Hatmi AMS, Brüggemann RJM, Melchers WJG, de Hoog S (2021). Molecular mechanisms of 5-fluorocytosine resistance in yeasts and filamentous fungi. J Fungi (Basel).

[27] Margulis L (1975). Symbiotic theory of the origin of eukaryotic organelles; criteria for proof. Symp Soc Exp Biol.

[28] Tzagoloff A, Foury F, Akai A (1976). Assembly of the mitochondrial membrane system. XVIII. Genetic loci on mitochondrial DNA involved in cytochrome b biosynthesis. Mol Gen Genet.

[29] Coruzzi G, Tzagoloff A (1979). Assembly of the mitochondrial membrane system. DNA sequence of subunit 2 of yeast cytochrome oxidase. J Biol Chem.

[30] Macino G, Tzagoloff A (1980). Assembly of the mitochondrial membrane system: sequence analysis of a yeast mitochondrial ATPase gene containing the oli-2 and oli-4 loci. Cell.

[31] Wang X, Ryu D, Houtkooper RH, Auwerx J (2015). Antibiotic use and abuse: a threat to mitochondria and chloroplasts with impact on research, health, and environment. Bioessays.

[32] Nosek J, Fukuhara H (1994). NADH dehydrogenase subunit genes in the mitochondrial DNA of yeasts. J Bacteriol.

[33] Sor F, Fukuhara H (1982). Identification of two erythromycin resistance mutations in the mitochondrial gene coding for the large ribosomal RNA in yeast. Nucleic Acids Res.

[34] Dujon B (1980). Sequence of the intron and flanking exons of the mitochondrial 21S rRNA gene of yeast strains having different alleles at the omega and rib-1 loci. Cell.

[35] Blanc H, Wright CT, Bibb MJ, Wallace DC, Clayton DA (1981). Mitochondrial DNA of chloramphenicol-resistant mouse cells contains a single nucleotide change in the region encoding the 3’ end of the large ribosomal RNA. Proc Natl Acad Sci.

[36] Wilson DN (2014). Ribosome-targeting antibiotics and mechanisms of bacterial resistance. Nat Rev Microbiol.

[37] do Valle Matta MA, Jonniaux JL, Balzi E, Goffeau A, van den Hazel B (2001). Novel target genes of the yeast regulator Pdr1p: a contribution of the TPO1 gene in resistance to quinidine and other drugs. Gene.

[38] Markovich S, Yekutiel A, Shalit I, Shadkchan Y, Osherov N (2004). Genomic approach to identification of mutations affecting caspofungin susceptibility in *Saccharomyces cerevisiae*. Antimicrob Agents Chemother.

[39] Albertsen M, Bellahn I, Krämer R, Waffenschmidt S (2003). Localization and function of the yeast multidrug transporter Tpo1p. J Biol Chem.

[40] Vögtle F-N, Burkhart JM, Gonczarowska-Jorge H, Kücükköse C, Taskin AA (2017). Landscape of submitochondrial protein distribution. Nat Commun.

[41] Dartier J, Lemaitre E, Chourpa I, Goupille C, Servais S (2017). ATP-dependent activity and mitochondrial localization of drug efflux pumps in doxorubicin-resistant breast cancer cells. Biochim Biophys Acta Gen Subj.

[42] Cunha-Oliveira T, Ferreira LL, Coelho AR, Deus CM, Oliveira PJ (2018). Doxorubicin triggers bioenergetic failure and p53 activation in mouse stem cell-derived cardiomyocytes. Toxicol Appl Pharmacol.

[43] Kumar A, Worobec EA (2005). HasF, a TolC-homolog of *Serratia marcescens*, is involved in energy-dependent efflux. Can J Microbiol.

[44] Huang J, O’Toole PW, Shen W, Amrine-Madsen H, Jiang X (2004). Novel chromosomally encoded multidrug efflux transporter MdeA in *Staphylococcus aureus*. Antimicrob Agents Chemother.

[45] Srinivasan VB, Venkataramaiah M, Mondal A, Rajamohan G (2015). Functional characterization of AbeD, an RND-type membrane transporter in antimicrobial resistance in *Acinetobacter baumannii*. PLoS One.

[46] Paulsen IT, Brown MH, Skurray RA (1996). Proton-dependent multidrug efflux systems. Microbiol Rev.

[47] Ammor MS, Flórez AB, Margolles A, Mayo B (2006). Fluorescence spectroscopy: a rapid tool for assessing tetracycline resistance in *Bifidobacterium longum*. Can J Microbiol.

[48] Shuvo SR, Wiens LM, Subramaniam S, Treberg JR, Court DA (2019). Increased reactive oxygen species production and maintenance of membrane potential in VDAC-less *Neurospora crassa* mitochondria. J Bioenerg Biomembr.

[49] Hassan KA, Liu Q, Elbourne LDH, Ahmad I, Sharples D (2018). Pacing across the membrane: the novel PACE family of efflux pumps is widespread in Gram-negative pathogens. Res Microbiol.

[50] Summers WAT, Wilkins JA, Dwivedi RC, Ezzati P, Court DA (2012). Mitochondrial dysfunction resulting from the absence of mitochondrial porin in *Neurospora crassa*. Mitochondrion.

[51] Kornelsen V, Kumar A (2021). Update on multidrug resistance efflux pumps in *Acinetobacter spp*. Antimicrob Agents Chemother.

[52] Du D, Wang-Kan X, Neuberger A, van Veen HW, Pos KM (2018). Multidrug efflux pumps: structure, function and regulation. Nat Rev Microbiol.

[53] Brown MH, Paulsen IT, Skurray RA (2002). The multidrug efflux protein norm is a prototype of a new family of transporters. Mol Microbiol.

[54] Paulsen IT, Brown MH, Dunstan SJ, Skurray RA (1995). Molecular characterization of the staphylococcal multidrug resistance export protein QacC. J Bacteriol.

[55] Eicher T, Brandstätter L, Pos KM (2009). Structural and functional aspects of the multidrug efflux pump AcrB. Biol Chem.

[56] Hassan KA, Liu Q, Henderson PJF, Paulsen IT (2015). Homologs of the *Acinetobacter baumannii* AceI transporter represent a new family of bacterial multidrug efflux systems. mBio.

[57] Saier MH, Reddy VS, Moreno-Hagelsieb G, Hendargo KJ, Zhang Y (2021). The transporter classification database (TCDB): 2021 update. Nucleic Acids Res.

[58] Dean M, Allikmets R (2001). Complete characterization of the human ABC gene family. J Bioenerg Biomembr.

[59] Procko E, O’Mara ML, Bennett WFD, Tieleman DP, Gaudet R (2009). The mechanism of ABC transporters: general lessons from structural and functional studies of an antigenic peptide transporter. FASEB J.

[60] Khunweeraphong N, Kuchler K (2021). Multidrug resistance in mammals and fungi-from MDR to PDR: a rocky road from atomic structures to transport mechanisms. Int J Mol Sci.

[61] Prasad R, Rawal MK (2014). Efflux pump proteins in antifungal resistance. Front Pharmacol.

[62] De Rossi E, Arrigo P, Bellinzoni M, Silva PAE, Martín C (2002). The multidrug transporters belonging to major facilitator superfamily in *Mycobacterium tuberculosis*. Mol Med.

[63] Drew D, North RA, Nagarathinam K, Tanabe M (2021). Structures and general transport mechanisms by the major facilitator superfamily (MFS). Chem Rev.

[64] Barabote RD, Thekkiniath J, Strauss RE, Vediyappan G, Fralick JA (2010). Xenobiotic efflux in bacteria and fungi: a genomics update. Adv Enzymol Relat Areas Mol Biol.

[65] Spatafora JW, Aime MC, Grigoriev IV, Martin F, Stajich JE (2017). The fungal tree of life: from molecular systematics to genome-scale phylogenies. Microbiol Spectr.

[66] Andrade AC, Van Nistelrooy JG, Peery RB, Skatrud PL, De Waard MA (2000). The role of ABC transporters from *Aspergillus nidulans* in protection against cytotoxic agents and in antibiotic production. Mol Gen Genet.

[67] Fernández-Aguado M, Martín JF, Rodríguez-Castro R, García-Estrada C, Albillos SM (2014). New insights into the isopenicillin N transport in *Penicillium chrysogenum*. Metab Eng.

[68] Martín JF (2020). Transport systems, intracellular traffic of intermediates and secretion of β-lactam antibiotics in fungi. Fungal Biol Biotechnol.

[69] Neupert W (1997). Protein import into mitochondria. Annu Rev Biochem.

[70] Fukasawa Y, Tsuji J, Fu SC, Tomii K, Horton P (2015). MitoFates: improved prediction of mitochondrial targeting sequences and their cleavage sites. Mol Cell Proteomics.

[71] Kreimendahl S, Schwichtenberg J, Günnewig K, Brandherm L, Rassow J (2020). The selectivity filter of the mitochondrial protein import machinery. BMC Biol.

[72] Morgenstern M, Stiller SB, Lübbert P, Peikert CD, Dannenmaier S (2017). Definition of a high-confidence mitochondrial proteome at quantitative scale. Cell Rep.

[73] Di Bartolomeo F, Malina C, Campbell K, Mormino M, Fuchs J (2020). Absolute yeast mitochondrial proteome quantification reveals trade-off between biosynthesis and energy generation during diauxic shift. Proc Natl Acad Sci U S A.

[74] Kispal G, Csere P, Guiard B, Lill R (1997). The ABC transporter Atm1p is required for mitochondrial iron homeostasis. FEBS Lett.

[75] Young L, Leonhard K, Tatsuta T, Trowsdale J, Langer T (2001). Role of the ABC transporter Mdl1 in peptide export from mitochondria. Science.

[76] Finn RD, Clements J, Eddy SR (2011). HMMER web server: interactive sequence similarity searching. Nucleic Acids Res.

[77] Altschul SF, Madden TL, Schäffer AA, Zhang J, Zhang Z (1997). Gapped BLAST and PSI-BLAST: a new generation of protein database search programs. Nucleic Acids Res.

[78] Keeping A, Deabreu D, Dibernardo M, Collins RA (2011). Gel-based mass spectrometric and computational approaches to the mitochondrial proteome of *Neurospora*. Fungal Genet Biol.

[79] Shuvo SR, Motnenko A, Krokhin OV, Spicer V, Court DA (2022). Proteomic shifts reflecting oxidative stress and reduced capacity for protein synthesis, and alterations to mitochondrial membranes in *Neurospora crassa* lacking VDAC. Microorganisms.

[80] Fernandes AS, Gonçalves AP, Castro A, Lopes TA, Gardner R (2011). Modulation of fungal sensitivity to staurosporine by targeting proteins identified by transcriptional profiling. Fungal Genet Biol.

[81] Pedro Gonçalves A, Silva N, Oliveira C, Kowbel DJ, Glass NL (2015). Transcription profiling of the *Neurospora crassa* response to a group of synthetic (thio)xanthones and a natural acetophenone. Genom Data.

[82] Lum PY, Armour CD, Stepaniants SB, Cavet G, Wolf MK (2004). Discovering modes of action for therapeutic compounds using a genome-wide screen of yeast heterozygotes. Cell.

[83] Knight JA, Courey AJ, Stebbins B (1982). Second-site antibiotic resistance mutations in the ribosomal region of yeast mitochondrial DNA. Curr Genet.

[84] Masuda T, Ito S, Ohtsuki S (2021). Advances in sample preparation for membrane proteome quantification. Drug Discov Today Technol.

[85] Young MJ (2017). Off-target effects of drugs that disrupt human mitochondrial DNA maintenance. Front Mol Biosci.

[86] Young CKJ, Wheeler JH, Rahman MM, Young MJ (2021). The antiretroviral 2’,3’-dideoxycytidine causes mitochondrial dysfunction in proliferating and differentiated HepaRG human cell cultures. J Biol Chem.

[87] Grigoriev IV, Nikitin R, Haridas S, Kuo A, Ohm R (2014). MycoCosm portal: gearing up for 1000 fungal genomes. Nucleic Acids Res.

[88] Vélez CG, Letcher PM, Schultz S, Powell MJ, Churchill PF (2011). Molecular phylogenetic and zoospore ultrastructural analyses of *Chytridium olla* establish the limits of a monophyletic Chytridiales. Mycologia.

[89] Spatafora JW, Chang Y, Benny GL, Lazarus K, Smith ME (2016). A phylum-level phylogenetic classification of zygomycete fungi based on genome-scale data. Mycologia.

[90] Kobayashi Y, Maeda T, Yamaguchi K, Kameoka H, Tanaka S (2018). The genome of *Rhizophagus clarus* HR1 reveals a common genetic basis for auxotrophy among arbuscular mycorrhizal fungi. BMC Genomics.

[91] Arnaud MB, Cerqueira GC, Inglis DO, Skrzypek MS, Binkley J (2012). The Aspergillus genome database (AspGD): recent developments in comprehensive multispecies curation, comparative genomics and community resources. Nucleic Acids Res.

[92] Galagan JE, Calvo SE, Borkovich KA, Selker EU, Read ND (2003). The genome sequence of the filamentous fungus *Neurospora crassa*. Nature.

[93] Goffeau A, Barrell BG, Bussey H, Davis RW, Dujon B (1996). Life with 6000 genes. Science.

[94] Jones T, Federspiel NA, Chibana H, Dungan J, Kalman S (2004). The diploid genome sequence of *Candida albicans*. Proc Natl Acad Sci U S A.

[95] Wood V, Gwilliam R, Rajandream M-A, Lyne M, Lyne R (2002). The genome sequence of *Schizosaccharomyces pombe*. Nature.

[96] Zhu Z, Zhang S, Liu H, Shen H, Lin X (2012). A multi-omic map of the lipid-producing yeast *Rhodosporidium toruloides*. Nat Commun.

[97] Kämper J, Kahmann R, Bölker M, Ma L-J, Brefort T (2006). Insights from the genome of the biotrophic fungal plant pathogen *Ustilago maydis*. Nature.

[98] Morin E, Kohler A, Baker AR, Foulongne-Oriol M, Lombard V (2012). Genome sequence of the button mushroom *Agaricus bisporus* reveals mechanisms governing adaptation to a humic-rich ecological niche. Proc Natl Acad Sci U S A.

[99] Lin J, Zhou D, Steitz TA, Polikanov YS, Gagnon MG (2018). Ribosome-targeting antibiotics: modes of action, mechanisms of resistance, and implications for drug design. Annu Rev Biochem.

[100] Mahler HR, Perlman PS (1971). Mitochondriogenesis analyzed by blocks on mitochondrial translation and transcription. Biochemistry.

[101] Davey PJ, Haslam JM, Linnane AW (1970). Biogenesis of mitochondria. 12. The effects of aminoglycoside antibiotics on the mitochondrial and cytoplasmic protein-synthesizing systems of *Saccharomyces cerevisiae*. Arch Biochem Biophys.

[102] Sulavik MC, Houseweart C, Cramer C, Jiwani N, Murgolo N (2001). Antibiotic susceptibility profiles of *Escherichia coli* strains lacking multidrug efflux pump genes. Antimicrob Agents Chemother.

[103] Cheng K, Ren L, Ki T (2010). Inhibitory concentrations of kanamycin in the presence of ppgpp synthase RelA confer protection against subsequent lethal antibiotic assaults in E. coli CP78. J Exp Microbiol Immunol.

[104] Lamb AJ, Clark-Walker GD, Linnane AW (1968). The biogenesis of mitochondria. 4. The differentiation of mitochondrial and cytoplasmic protein synthesizing systems in vitro by antibiotics. Biochim Biophys Acta.

[105] François B, Russell RJM, Murray JB, Aboul-ela F, Masquida B (2005). Crystal structures of complexes between aminoglycosides and decoding A site oligonucleotides: role of the number of rings and positive charges in the specific binding leading to miscoding. Nucleic Acids Res.

[106] Hughes AR, Wilkie D (1972). Genetic analysis of mitochondrial resistance to tetracycline in *Saccharomyces cerevisiae*. Heredity (Edinb).

[107] Wolf K, Dujon B, Slonimski PP (1973). Mitochondrial genetics. V. Multifactorial mitochondrial crosses involving a mutation conferring paromomycin-resistance in *Saccharomyces cerevisiae*. Mol Gen Genet.

[108] Mehta R, Champney WS (2003). Neomycin and paromomycin inhibit 30S ribosomal subunit assembly in *Staphylococcus aureus*. Curr Microbiol.

[109] Islam MD (2021). Investigating several putative mitochondrial pumps in Saccharomyces cerevisiae, MSc Thesis.

[110] Morgenstern M, Peikert CD, Lübbert P, Suppanz I, Klemm C (2021). Quantitative high-confidence human mitochondrial proteome and its dynamics in cellular context. Cell Metab.

[111] Lee CP, Eubel H, Solheim C, Millar AH (2012). Mitochondrial proteome heterogeneity between tissues from the vegetative and reproductive stages of *Arabidopsis thaliana* development. J Proteome Res.

[112] Wirsing L, Klawonn F, Sassen WA, Lünsdorf H, Probst C (2015). Linear discriminant analysis identifies mitochondrially localized proteins in *Neurospora crassa*. J Proteome Res.

