# Supplementary material for: Do mitochondria use efflux pumps to protect their ribosomes from antibiotics?
Source: Microbiology (Reading). 2023 Jan 18;169(1):001272. doi: 10.1099/mic.0.001272 (PMC9993110; doi:10.1099/mic.0.001272)
Supplement: Supplementary material 2 [file mic-169-1272-s001.pdf]

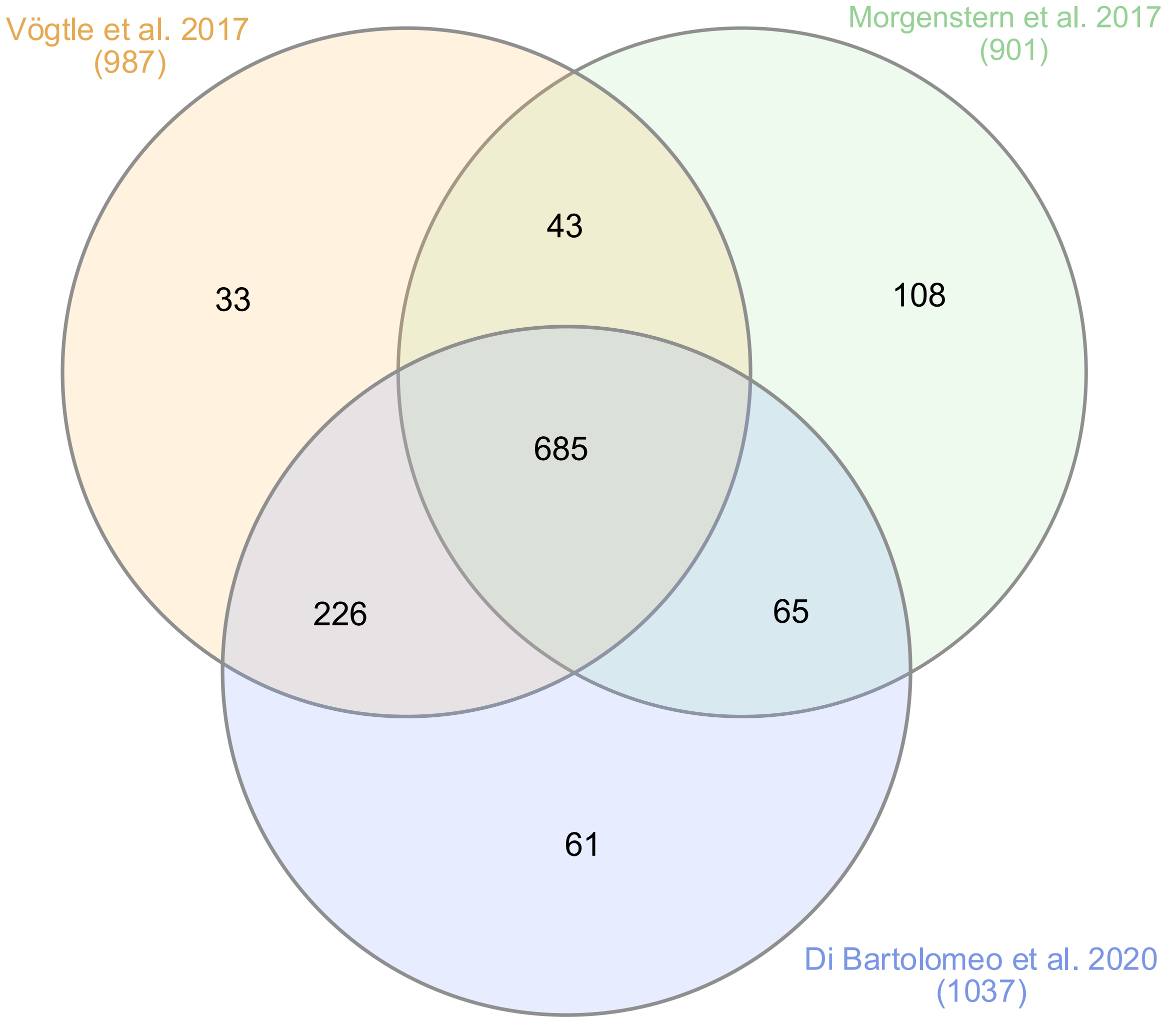

Figure 1. Venn diagram comparing the identified proteins associated with the mitochondrion in three separate mitochondrial proteome studies in *S. cerevisiae.*
